# Work–Life Balance and Mental and Physical Health among Warsaw Specialists, Managers and Entrepreneurs

**DOI:** 10.3390/ijerph20010492

**Published:** 2022-12-28

**Authors:** Agnieszka A. Borowiec, Wojciech Drygas

**Affiliations:** Department of Epidemiology, Cardiovascular Disease Prevention and Health Promotion, The Cardinal Stefan Wyszynski National Institute of Cardiology, 04-628 Warsaw, Poland

**Keywords:** work–life balance, middle class, self-rated health, mental health, Poland

## Abstract

Many studies have shown that low work–life balance (WLB) can be harmful to health. Poland is a country with one of the lowest indicators on the WLB scale among European countries but there are only a few studies about the connection between WLB and health. The present analysis aimed to answer the questions of whether the lower WLB among Warsaw’s middle class correlates with poorer mental and physical health, and what life orientations and values typical of the middle class are related to work–life balance. Two surveys were conducted in the years 2003 and 2013 on the quota samples of 500 members of the Warsaw middle class: specialists, managers, and entrepreneurs. The current analysis has indicated the connection between a lower level of WLB and worse mental and physical health. Some middle-class life orientations are connected with a high WLB. The relationship between WLB and health was stronger in 2013 than in 2003. It can be considered a result of mentality and lifestyle changes and generational renewal. The study should be repeated in 2023 after the COVID-19 pandemic as the work situation of the middle class may have changed.

## 1. Introduction

Changes taking place in the modern world have caused the problem of balance (or its lack of) between occupational work and other areas of life to become increasingly important. One of the most important consequences of low work–life balance can be worsening physical and mental health [1,2].

Poland is still a country in rapid social and economic transition, and its citizens are in worse health condition than people in the majority of European countries. Health indicators such as life expectancy, and premature mortality and morbidity are worse than in the Western Europe countries. Both objective measures and people’s health self-assessment indicate a poorer health condition of Poles than people in other parts of Europe [3,4]. Although work–life balance indicators in Poland are lower than in the majority of societies in Europe [1,2], there is little research in Poland about the connection between WLB and health.

Work–life balance can be defined as a kind of relationship between the social roles fulfilled in occupational work and in personal life assessed by individuals as being satisfactory, conflict-free, harmonious and enabling efficient functioning in both private and work areas [5]. In more complex definitions, the following have also been pointed out: the distinction between balance and conflict that can be treated as separated dimensions as it is possible to experience both of them at the same time [6]; the existence of various aspects/dimensions in which the conflict/balance takes place, e.g., time, behaviour, involvement, satisfaction [7,8,9]; direction of conflict: work–life conflict takes place when the professional roles interfere with non-professional roles, and life–work conflict arises when non-professional roles interfere with the professional roles [10]; the consequences of work and life interplay that can be positive or negative [5,11].

Among the negative consequences of low work–life balance are worsening physical and mental health. There are two probable factors that cause low work–life balance to contribute to worsening health: stress and poor time management [10,12]. Conflicting demands of occupational work and personal roles lead to stress as they disrupt the balance between the individual and his or her environment [13,14]. In turn, long-term stress leads to physiological processes damaging particular parts of the body or systems and, consequently, promoting physical disorders and diseases. According to the job demand–resources theory [15], employee stress and adverse health result from a lack of balance between high job demands and low job resources. Additionally, it can be accompanied by attempts to reduce the high level of strain by unhealthy behaviours such as tobacco smoking or eating sweets or salty snacks [12,16,17,18]. Poor time management can be a consequence of lack of time for/or lack of involvement in doing healthy or avoiding unhealthy behaviour.

As has been indicated by many studies, people with a higher conflict or lower balance between work and life are more likely to report worse mental and physical health, to suffer from health ailments and to have worse health parameters [6,12,19,20,21,22,23,24,25,26,27,28,29]. Although Poland is a country with one of the lowest indicators on the WLB scale among European countries [1,2] there are few studies researching the connection between WLB and health. These have shown that Polish people who have lower levels of work–life balance are more likely to have poorer physical and mental health [30,31,32]. However, these studies were conducted on relatively small and special samples, and while they indicate that the problem of relationships between work–life balance and health is important, but this needs to be further examined and analysed.

Warren [33] has stated that discourse on work–life balance concerns mainly the middle class neglecting the situation of the working class. The reason is that researchers pay much more attention to the work–life conflict/balance taking place because of extended working hours (which occurs more often in mental work than in manual work), than to other aspects of work. Indeed, many studies devoted to the problem of work–life balance have focused on people working in positions that are ascribed to the middle class, in narrow or broad terms [34,35,36,37,38,39,40,41,42,43,44,45,46,47,48,49]. But there are also other middle-class characteristics causing the problem of work–life balance that could be focused on by researchers. Middle class positions in the social structure are defined by amount and source of income i.e., possession of the material property, skills, qualifications or credentials (market position), a work situation connected with doing non-manual tasks and a lifestyle expressing this social status [50,51,52,53]. The special set of values and life orientations including self-discipline, regularity, diligence, consent to delayed gratification and individualism, with the most important role played by occupational work, have been attributed to the middle class, especially in developing countries as referred to by [54,55], and which is still studied as a Protestant Work Ethic in many different societies [56,57,58]. Achieving a high social position through occupational work, the great complexity of the professional duties, diligence in doing a job, a high level of time and mental involvement in occupational duties as well as status-oriented consumption that requires adequate funding [52,59,60,61,62] can cause middle-class members to be highly exposed to low work–life balance. The middle class is also known as a social category leading special lifestyles containing healthy practices [63,64,65,66,67,68].

Based on the most influential opinions about the existence of a middle class in Poland after 1989 [69,70,71,72,73,74,75,76,77] it has been assumed that it exists in some form and consists of small and medium entrepreneurs, professionals with university degrees and managers [71,75]. Some studies have indicated its similarities to the middle class in developed countries; work ethos, consumerism and healthy practices not only increase in Polish society, but they are also more common in social categories included in the middle class [78,79,80,81,82,83,84].

According to a study by the Institute of Management [85], the categories of people that are included in the middle class are very exposed to the lack of work–life balance. On the contrary, the results of the third Korean Working Conditions Survey show that people working in middle-class positions are less likely to have a low work–life balance than categories occupying higher or lower positions in the social structure [19].

Thus, the present analysis has two purposes. The first one is the confirmation of relationships between low work–life balance and poor indicators of mental and physical health in Poland. The few studies conducted to date have involved small samples and narrow populations. Therefore, the aim of the analysis will be to identify the correlation between the state of health and the level of work–life balance among the middle class at two time points: in 2003 and 2013. The result will be a valuable supplement to the findings obtained so far. The second aim of the analysis is an attempt to find those middle-class characteristics that correlate with work–life balance. Some of the values and life orientations focused on occupational work, such as individualism or tendency to behave in accordance with a Protestant Work Ethic, as well as the special approach to health seem to cause middle-class members to be especially exposed to conflict between occupational work and private life. The research questions are:

1. Does low work–life balance among middle-class members cause their physical and mental conditions to be worse?
2. What characteristics and, especially, what kind of life orientations and values ascribed typically to the middle class, correlate with the level of work–life balance among people located in middle-class positions?

The following hypotheses were made:

1. The lower the work–life balance, the poorer the physical and the mental health.
2. The higher the level of life orientations typical of the middle class, the lower the work–life balance.

## 2. Materials and Methods

### 2.1. Study Population and Samples

Two studies using personal interviews were conducted in the years 2003 and 2013 on two Warsaw middle-class quota samples of 500 people. The studied population consisted of small and medium entrepreneurs, specialists with university degrees, and managers. The frequency distributions of characteristics such as gender, education, occupational position, sector of industry, and the district of living in Warsaw were used for the sample construction. They were taken from the results of the research carried out on the randomly selected representative samples of Warsaw inhabitants performed by the research centres TNS OBOP (2003) and CBOS (2013), as well as the statistical publications *Panorama dzielnic Warszawy w 2000 roku* (Panorama of Warsaw districts in 2000) [86], *Rocznik statystyczny Województwa Mazowieckiego 2002* (Statistical Yearbook of Mazowieckie Voivodships) [87], *Panorama dzielnic Warszawy w 2011 roku* (Panorama of Warsaw districts in 2011 [88], and *Barometr Warszawski 2013* (Warsaw Barometer 2013) [89].

### 2.2. Work–Life Balance Measurement

In our analysis, WLB was understood as the subjective appraisal that fulfilling occupational roles does not interfere with personal roles. An accent was put on how the non-work domain is interfered with by work duties. Nevertheless, the distinction between work–life conflict/balance and life–work conflict/balance can be ignored as they are very closely related: people who experience work–to–non-work conflict are also more likely to report non-work–to–work conflict [5]. We have focused on if, and how, time devoted to the occupational work interferes with time that should be sacrificed to family and personal activities; however, total separation of these aspects from the other dimensions in which WLB takes place (sucha as involvement or behaviour) is impossible. The balance and conflict were treated as extremes of the same continuum, not two separate dimensions.

Work–life balance (WLB) was measured using a scale that included 6 items referring to the relationship between work and different areas of personal life. Respondents were asked the multi-item question: “To what extent do the following statements reflect your approach to occupational work. I will read them one by one, and I will ask you, to what extent you agree with each of them.” The statements are as follows:

- I sometimes give up going to the cinema, theatre, or social gathering to do my professional work during this time.
- Even in situations of overloading, I find time for physical activity.
- I usually find enough time for my family.
- I am often so busy that I do not pay attention to what I eat and when.
- It happens that I do not use sick leave, even when I am really sick and I feel bad.
- I have enough time to be able to pursue my non-professional interests or hobbies.

Respondents answered the question using a five-point Likert scale: “I definitely agree”, “I rather agree”, “I rather disagree”, “I strongly disagree”, and “difficult to say”. A WLB scale was calculated by summing points ascribed to these answers. The answer “difficult to say” was inserted in the middle of the scale with a value of 3. In the case of the statements expressing lack of enough time for personal activities due to occupational work, a value of 1 was ascribed to the answer “I definitely agree”, and a value of 5 to the answer “I strongly disagree”. In the case of the statements indicating that given person has enough time for personal activities in spite of occupational duties, a value of 5 was ascribed to the answer “I definitely agree”, and a of value 1 to the answer “I strongly disagree”. After summing, the scale takes values from 1 to 5. The higher the value, the greater the balance between work and other spheres of life. Cronbach’s Alpha reliability ratio is 0.665 for the 2003 data and 0.593 for the 2013 data. These values are not very high, especially for the data from 2013 (slightly under 0.6) but this appears to be a result of the rather small number of statements included in the scale, and the fact that these statements relate to different activities (with different importance for the people) competing with occupational work. Thus, these values of Alpha seem to be permissible. We wanted also to gain comparable results in both years 2003 and 2013 and this would not have been possible without including the same statements in both scales.

### 2.3. Mental and Physical Health Measurements

The answer to the question about health self-assessment in comparison with other people of the same sex and age as well as declarations of not having serious disease diagnosed by a doctor were treated as indicators of physical health. Health self-assessments can be used to provide information about people’s real health conditions as the connection between health self-assessment and the state of health has been confirmed in many studies [90,91,92,93]. The health self-assessment has been used as a measure of health also in other studies aimed at discovering the relationship between work–life balance and health [24,94]. To measure mental health the selected subscales of Occupational Stress Indicator-2 (OSI-2; created by C. L. Cooper and others, adapted to Polish conditions by Widerszal-Bazyl 2001 [95]) were used. They are as follows: “dynamism”, “satisfaction and peace of mind” and ”peace and energy”. The “satisfaction and peace of mind” scale relates to emotions such as anxiety, self-confidence, helplessness or sadness. The “dynamism” scale expresses readiness to act. The “peace and energy” scale contains items relating to the physical symptoms such as unexplained fatigue and exhaustion, a tendency to eat, drink or smoke more than usual, shortness of breath or dizziness, muscle twitching, a feeling of stinging or pain in some parts of the body for no apparent reason, and a feeling of reluctance to get up in the morning. The scales were created based on eighteen questions about the psychological and physical symptoms of stress. The scores for each person were calculated using special formulas [95]. A higher score on the scale means lower stress, i.e., better mental health.

### 2.4. Life Orientations Measurements

Life orientations: Protestant Work Ethic (PWE) and individualism were measured by the scales used previously by Domański and his associates [72,85,96,97] to study different aspects of the mentality of the Polish society to identify the middle class in Poland.

The tendency to behave according to the Protestant Work Ethic was measured using five questions formulated by Domański [85]. Choosing a lower-paid but guaranteed job, systematically saving a part of one’s salary, preferring a systematic and certain profit instead of taking risks, choosing investment in the development of one’s own company rather than spending money on one’s own needs and pleasures and deciding to work even if it is not financially necessary were considered indicators of a tendency to behave in agreement with a Protestant Work Ethic. The study also included two types of individualism: “self-reliance” and “orientation towards success and career”. “Self-reliance” was measured by items selected from the I-E. Rotter’s scale (originally used for measuring the locus of control) and adapted to the Polish situation by Titkow [14]. These items concern a feeling of having little influence over one’s own life, an opinion that “what is going to happen, will happen”, the opinion that if they are defeated it is not their fault, and the opinion that the majority of one’s problems are caused by fate or circumstances. Disagreement with these points pointed to a high level of self-reliance. “Orientation towards success and career” was measured by five questions formulated by Titkow and used for measuring the fear of success [98]. The sentences concerned opinions about somebody being unpopular through being successful at work, the feeling that people who achieve success often feel sad and lonely, preferring not to show one’s abilities rather than to be seen as swaggering or showing off, and the feeling that people give far more in the pursuit of success than the reward of success will ever repay them. Disagreement with these sentences pointed to a high level of this kind of individualism. Respondents answered the questions about individualism using a Likert scale.

### 2.5. Statistical Analyses

Statistical analyses were performed separately for data obtained in 2003 and 2013. Analyses using the logistic regression method were performed (using the enter method, with the probability of entry being 0.01 and probability of removal being 0.05) to identify the connection between the work–life balance (as an explanatory variable) and physical health. Multiple linear regression analyses were employed to uncover the relationships between the work–life balance and the dimension of psychological health (using the backward method with the probability of entry being 0.01 and probability of removal being 0.05). These models of regressions were counted for every dependent variable related to the status of health. The socio-demographic characteristics and characteristics regarding occupational work (including the working time) were put into these models as controlled variables.

A multiple linear regression analysis using the backward method with the probability of entry at 0.01 and the probability of removal at 0.05 was calculated to explain the level of work–life balance. The socio-demographic variables (gender, age, education, marital status, number of people in the household, having children under 18 years of age living in one household), variables connected with the work situation (professional status, doing extra paid work, willingness to change work, legal form of the workplace, working time) and variables expressing life orientations such as individualism, Protestant Work Ethics and values–“family and children”, “occupation and work”, “spare time and rest”, “friends and acquaintances”, “relatives”, “religion and church”, “politics and public life”, “neighbour”, “health”–were inserted into the model.

## 3. Results

**Figure 1:**
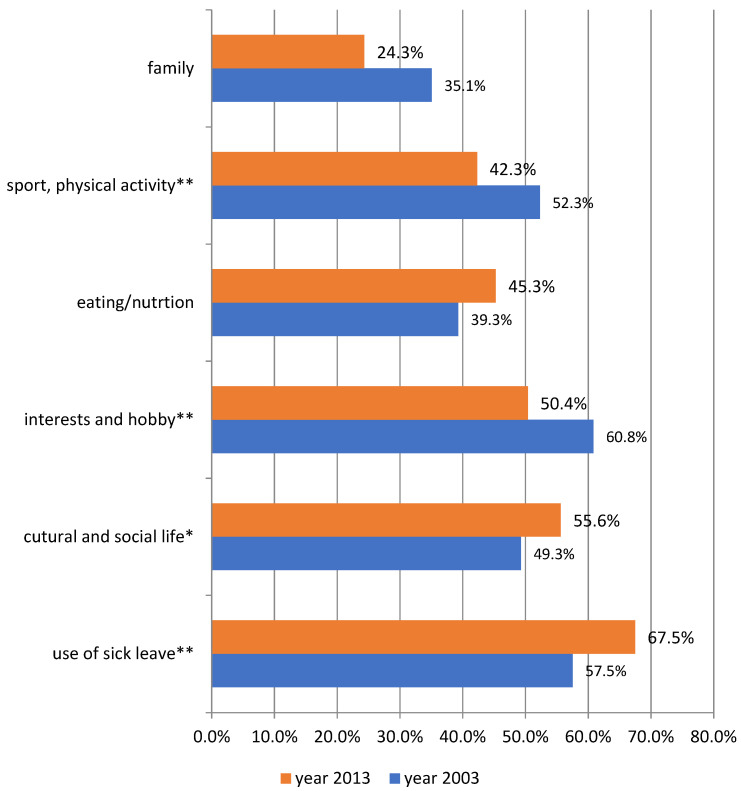
Declaration of lack of enough time to be active in selected spheres of life due to overloading by occupational work depending on the year of the survey (percentage of answers “I definitely agree” and “I rather agree” in the case of statements indicating lack of enough time for non-professional activities, and percentage of answers “I rather disagree” and “I strongly disagree” in the case of answers indicating that he or she always has enough time for non-professional activities). The significance of the differences between 2003 and 2013 was tested using the Chi-square test. * *p* ≤ 0.05; ** *p* ≤ 0.01.

Figure 1 presents the percentages of people whose responses indicate that they do not maintain a satisfying balance between occupational work and life in different areas. In 2003, about 60% of respondents declared that they did not have enough time to pursue their hobbies and interests because of excessive work (61%) and that they sometimes did not use sick leave despite feeling unwell (58%). Approximately every second respondent stated that sometimes he or she did not find enough time for physical activity because of work overloading (52%), and had given up social meetings or cultural participation because of professional duties (49%). Two-fifths did not pay attention to what they ate and when, because they were so busy (39%), and about every third declared that they did not find time for their family (35%). Distributions of answers obtained in the second study, i.e., in 2013, show that most often the respondents declared not using sick leave: 68% of them gave up sick leave sometimes because of occupational work despite feeling unwell. Fifty per cent declared that they had no time to pursue their non-professional interests and hobbies and even more of them had no time for cultural and social life (56%). Almost half did not find time to undergo healthy behaviour, such as taking care of their diet (45%) or practising sport (42%). In the case of 24% of respondents time needed for doing professional work was in conflict with time devoted to the family.

There are differences between the respondents’ declarations in 2003 and 2013. In 2013, they were less likely to declare a lack of time for their family, practising sports and pursuing their hobbies despite excess work, but they were more likely to refrain from using sick leave and to forgo time for their cultural and social life. The values on the W-LB scales were 3.02 in 2003 and 3.06 in 2013 (Table 1). However, although the differences between the balance in particular aspects turned out to be statistically significant, there are no differences between the averages of the scales of the work–life balance in 2003 and 2013. The T-test was not statistically significant.

### 3.1. Work–Life Balance and Health

The relationship between the low level of balance between work and private life and negative health effects has been confirmed by the results of the present study. Although the relationship between physical health and work–life balance was observed only in 2013, a relationship between mental health and work–life balance existed in both 2003 and 2013. In 2013, an increase in work–life balance by 1 point increased the likelihood of assessing one’s health as better than other people’s health by 77%. Similarly, the likelihood of not having a chronic disease diagnosed by a medical doctor increased by 32% if the work–life balance increased by 1 point. These results are presented in Table 2.

Two dimensions of the occupational-stress scales used as measures of mental health—“satisfaction and peace of mind” and ”peace and energy”—were found to be correlated with the level of work–life balance. The higher the balance, the higher the level of mental health. Only the ”dynamism” scale proved to be unrelated to the work–life balance. (see Table 3). It was found that the relationships between the measures of mental health—the “satisfaction and peace of mind” and the “peace and energy” scales—and the work–life balance were greater in 2013 than in 2003. To sum up: all statistically significant relationships have indicated that the higher the work–life balance, the better the mental and physical health. These relationships were observed even if they were controlled by the time devoted to professional work and other characteristics.

### 3.2. Determinants of Work–Life Balance among Middle Class Members

#### 3.2.1. Family and Work Characteristics

The predictors of work–life balance among middle-class members are shown in Table 4. No connections between socio-demographic and family characteristics and work–life balance were observed except for the relationship between a higher level of education and greater work–life balance in 2003. However, some of the work characteristics were connected with the level of WLB. In 2013, owners were more exposed to the conflict between occupational work and life than specialists. This appears to be a result of their higher involvement in being in business. This connection was not observed 10 years earlier. In 2003, the higher work–life balance was related to the lower likelihood of declaring a willingness to change jobs; probably a lower level of work–life balance causes people to think about changing their job. Both in 2003 and in 2013, people devoting less time to professional work as well as those who had holidays lasting no shorter than seven days during the last twelve months had a higher work–life balance.

#### 3.2.2. Life Orientations and Values

The determinants of work–life balance were sought in life orientations typical of the middle class: two kinds of individualism and Protestant work ethics as well as the degree of appreciation of values such as “family and children”, “occupation and work”, “spare time and rest”, “friends and acquaintances”, “relatives”, “religion and church”, “politics and public life”, “neighbour”and “health”.

It was found that, in 2003, greater balance was positively associated with Protestant Work Ethics, while in 2013, higher self-reliance and orientation towards success and career were conducive to greater WLB.

The results showed also that, both in 2003 and 2013, the appreciation of some values was connected with the level of balance between work and private life. People who valued spare time and rest more had a higher balance between work and private life, while people who valued professional work more had a lower balance. Appreciation of “health” played a role only in 2013 when people who valued health more achieved a better work–life balance. In 2003, appreciating “neighbours” was connected with a higher work–life balance.

## 4. Discussion

The relationships between WLB and mental health, expressed as a low level of occupational stress, and physical health, as a positive health self-assessment and lack of serious disease diagnosed by a doctor, were observed among the Warsaw middle class, although correlations between physical health and WLB were significant only in 2013.

It confirms the results obtained in many previous studies. They indicate that people with a higher conflict or lower balance between work and life are more likely to report not only stress but also emotional exhaustion, low level of wellbeing (which includes positive mood, vitality and general interests), as well as depression, negative emotions (such as anger, anxiety, frustration and resentment), sleep problems, and mental exhaustion [6,19,20,21,22,23,24,25]. Poorer self-rated health turned out to be connected with a higher work–life conflict or a lower work–life balance in different populations [19,24,25,26]. People who suffer from a lack of work–life balance more often report health ailments such as musculoskeletal disease, headache/eyestrain, fatigue [19,28] and physical exhaustion [21]. The lack or low level of balance between work and life is also connected with worse health parameters such as high cholesterol, high body-mass index, the incidence of hypertension and worse physical stamina [12,20]. A study carried out among Swedish private and public primary healthcare employees [29] uncovered a positive relationship between a higher balance between work and life, and a better state of health, understood in terms of Salutogenesis as physical, mental and social well-being. The study conducted in Poland has also shown that people who have a lower level of work–life balance are more likely to have poorer physical and mental health. Both the work–family conflict and family–work conflict was found to be associated with poorer physical and mental health among 567 nurses employed in 21 Polish hospitals [30].

These relationships between WLB and health were observed in the present study even if they were controlled by the time devoted to professional work. This means that not only real time spent doing occupational tasks, but also people’s subjective assessment to what degree their occupational work disrupts fulfilling personal and familial duties can influence health. Nevertheless, extended working time turned out to be one of the predictors of low work–life balance. According to some other studies, average working time employed as a measure of WLB has been correlated with health. This connection has been observed by Chirkowska-Smolak [31]. Among 153 employees, people who declared working extended hours more often suffer from mental fatigue, physical exhaustion, frequently-occurring nervous irritability, sleep problems, emotional exhaustion, burnout, frequent infections, chronic headaches, anxiety attacks, stomach/duodenal ulcers and increased blood pressure. In addition, Rasmus et al. [32] have shown the correlation between longer average working hours per month and worse physical health among 129 employees belonging to the emergency medical staff.

The relationships between work–life balance and health became stronger (mental health) and statistically more significant (physical health) between 2013 and 2003, although the level of the work–life balance did not change and the state of health had deteriorated only slightly. It suggests that the meaning attributed to this situation had evolved. At the beginning of the Systemic Transformation of 1989 middle class members were prone to hard work and sacrifice their private life for occupational work while waiting for the benefits in the future, following a Protestant Work Ethic that is still an important characteristic of the middle class in developing countries [54,55,56,57,58]. With time, they have started to have higher expectations, probably as a consequence of increasing consumer aspirations [99]. Asceticism and the work ethos characterizing the “up-and-coming” societies began to be replaced or followed by the expectations to ”cut off coupons” and discountings high professional position and income by going on holiday abroad, participating in cultural and social life, and investing in health. Leszkowicz-Baczyński [100] lists seven consumer facilities that are the subject of the aspirations of the middle class emerging in Poland after 1989, including: spending time abroad; buying apartments, flats, houses and estates; and pursuing a healthy lifestyle, which can be manifested by the purchase of recreational areas.

The changes taking place in Poland were accompanied by the generation renewal in Europe and USA. The Baby Boomer Generation (people born between 1946 and 1964) has been displaced by the X and Y Generations (people born in the years 1965–1980 and 1981–1999, respectively) in the labour market. This displacement may result in a decline in willingness to sacrifice one’s own private life for occupational work among employees, and can have an impact on the conviction of how much time they will be prone to devote to both occupational work and private life. Compared to the Baby Boomers, the X and Y Generations have been characterized as those who change jobs more often and emphasize the need to maintain a balance between private and family life [101,102]. They also less strongly believe that hard work is a way to achieve success, are less convinced of the importance of occupational work in itself, less strongly condemn wasting time and are less likely to put much effort in to gain the rewards which will be obtained in the future [103]. The difference in perception of WLB between consecutive generations was shown by a study of medical doctors [40] that indicates that doctors born before the 1980s perceive their work–life balance to be better than those doing a similar job who were born later.

Stronger relationships between health and the work–life balance in 2013 than in 2003 could be also explained in terms of the model proposed by Rantanen et al. [5]. It includes four types of work–life balance that are based on the intersecting dimensions of enhancement and conflict resulting from owned resources, demands and gained rewards. These types are as follows: beneficial, harmful, passive and active. The beneficial type of balance is a consequence of a situation where there is no conflict between work and personal life, while there is a work–life enhancement experience. This occurs because the resources provided and gains attained from participation in multiple roles surpass the requirements. When there is a work–life conflict and there is no positive interaction (i.e., the demands related to performing roles exceed the obtained benefits) a harmful type of balance appears. The passive type of balance is created in a situation when there is neither conflict nor positive spillover between work and the non-professional sphere—the demands arising from the roles played as well as the rewards obtained are low. The active balance appears when the individual is very engaged in performing his/her roles, playing them both as a choice (“the will to succeed and achieve happiness in different life spheres”) and a necessity (because of demands related to these roles). This type of balance is characteristic of high demands and high benefits taking place simultaneously.

It can be argued that, in 2003, the members of the middle class had an active type of balance because they perceived playing the conflicting roles not only as a necessity but also as a choice. They were inclined to work hard and devote much time to occupational work at the cost of private life while waiting for benefits in the future. Ten years later, people expected the rewards for the same efforts to be larger and come earlier. They might have perceived their effort and sacrifices to be rewarded less than in 2003 due to the increase in their consumption aspirations and generational renewal, while also being under more pressure because of global economic crisis of 2008, and the processes of income polarization and precarisation [104,105,106,107]. It makes their situation closer to the harmful type of work–life balance than the active one. As a consequence, it can result in higher stress and poorer physical health. Analysis conducted on samples of Finnish university professionals and Finnish managers has indicated that people with the active type of work–life balance are more likely to report better health and to be less stressed or job exhausted than people representing the harmful type, although they both suffer from a similar level of work–life conflict [5].

Life orientations ascribed to the middle class mainly at the stage of its forming express the specific internal attitude to the occupational work that causes middle-class members to be more likely to perceive time devoted to occupational work as less conflicting with time devoted to other areas of life. Being more success and career-oriented, relying on oneself and tending to follow PWE cause middle class members to represent the active type of work–life balance more than the harmful one [5].

To sum up, the high levels of life orientations typical of the middle class seem to make people less exposed to low WLB and, consequently, to protected against worse physical and mental health. These orientations are deeply internalized and cause middle-class members to perceive their overload by work as a choice connected with waiting for benefits in the future, rather than a necessity resulting from the worsening economic situation or being in danger of lowering their social status.

It can be seen as contradictory that WLB was negatively connected at the same time as having a high appreciation for occupational work. The explanation is that these factors probably relate to different phenomena, despite ostensible similarities. Life orientations concern the internal tendency to estimate, recognise and behave in a particular way in relation to different phenomena [108]. Orientation towards success and career, self-reliance and a Protestant work ethic appear to be deeply internalized and to be expressed in different areas of activity such as work and private life. Meanwhile, declarations that value “occupation and work” as being very important or important can be superficial, and refer to the outer aspects of work such as necessity of keeping a job, earning money, maintaining socio-economic status or upholding one’s own positive image, but not to occupational work itself. People who value highly “leisure and rest” as well as “health” (in 2013 only) have a greater work–life balance, which suggests that people’s behaviour in these areas are in accordance with declared values.

The problem of the work–life balance and its healthy consequences among middle-class members needs to be further studied after the COVID-19 pandemic. As Frąckowiak-Sochańska [109] wrote, the increasing rate of people doing their work remotely during the COVID-19 pandemic (at the end of 2020, 11 percent of workers worked remotely) has intensified the processes of blurring the spatial, temporal and mental borders between occupational and private areas. It has led to the conflict of obligations and has caused establishing new relationships between occupational work and private life to be necessary. Fulfilling the occupational and family roles in this situation leads to negative psychological consequences such as emotional difficulties connected with over-loading, loss of agency and lack of verified ways of action. However, other studies [110] indicate positive effects of remote work for health. Furthermore, some studies on the Protestant Work Ethic or generational renewal suggest that trends observed in the study will continue [111,112]. As a result, the long-term consequences of changing way of work need to be studied, especially in the context of the disadvantageous health situation of Poles [3,4].

The strong point of this study is the possibility to estimate the relationships between work–life balance and state of health in two points of time separated by one decade. Furthermore, the study has investigated the connection between WLB and health that has been little recognised in Poland and other Central and Eastern European countries. Attempts were made to find characteristics common for the social categories included in the middle class which influence their work–life balance.

## 5. Conclusions

Lower work–life balance among the middle class in Warsaw was connected with worse mental (in 2003 and 2013) and physical health (in 2013) which supports the findings from earlier studies. The increasing strength of the relationship between work–life balance and the dimensions of mental health between 2003 and 2013 and the significant correlations between work–life balance and physical health in 2013, can be explained by changes of the mentality and lifestyle of Polish middle class and generational renewal in Europe and USA, causing the people’s perception of work situation and their expectations to change

Contrary to the hypothesis that higher levels of middle-class life orientations do not cause its members to be more exposed to a low work–life balance, it appears rather to protect them against poor WLB. These orientations can be treated as a resource which allows people to cope with work–life conflict, to derive more satisfaction from fulfilling occupational roles and to be in better health. It draws the attention towards the problem of work–life balance among the social groups for whom occupational work is only an obligation.

## 6. Limitation and Future Research

The study has some limitations. The work–life balance was measured using the unidimensional scale related to the balance or conflict between the time demands of performing occupational duties and fulfilling personal activities. It has not taken into account other dimensions of WLB although these aspects appear in some WLB concepts.

The study was restricted to the Warsaw’s middle class. Because of the lack of lists of people belonging to this population a randomly selected sample could not be used, instead the quota sample was employed. The limitation of the study to the Warsaw middle class leads to the question if the results of the study can be generalized to middle class in other parts of Poland and middle class in general. The findings should be interpreted carefully. Nevertheless, Warsaw is the capital city of Poland, one of the richest cities in Poland, and Warsaw’s middle class can be treated as a touchstone of shaping middle class in Poland what allows supposing that similar findings would be gained if the study contain middle class in other parts of Poland. However, taking into account the dissimilarities resulting from different sizes of places or regions of living in the country this supposition should be examined.

It is planned to conduct the next study about ten years after the second study with aim of measuring the changes in the level of work–life balance and its connection with mental and physical health among middle-class members as well as the relation between the level of WLB and such middle-class characteristics as special life orientations and values. In optimal, this study will be done on a representative sample of Polish middle class containing entrepreneurs, specialists with university degrees, and managers with Warsaw’s overrepresentation. In light of the current findings, it can be expected that the relationship between WLB and health will be higher than in 2013. Other factors that can influence WLB level and its connection with health are changes in work organization after COVID-19 pandemic and the economic crisis connected with the pandemic and The War on Ukraine.

## Figures and Tables

**Table 1:** Descriptive statistics.

2003					
	N	Mean	Std. Deviation	Minimum	Maximum
Work–life balance	475	3.02	0.79	1	5
”Satisfaction and peace of mind” (mental health)	499	27.75	6.10	8	42
“Dynamism” (mental health)	500	18.46	2.17	10	24
“Peace and energy” (mental health)	495	24.31	6.04	7	35
	N	Yes (%)	No (%)		
Positive health self-assessment—being in better health than other people of the same age and gender (physical health)	475	30.10	69.90		
Not having any chronic diseases or ailments diagnosed by a doctor (physical health)	498	80.70	19.30		
**2013**					
	**N**	**Mean**	**Std. Deviation**	**Minimum**	**Maximum**
Work–life balance	492	3.06	0.84	1	5
”Satisfaction and peace of mind” (mental health)	486	28.23	7.17	8	42
“Dynamism” (mental health)	496	18.41	2.33	10	25
“Peace and energy” (mental health)	492	23.45	5.99	4	35
	N	Yes (%)	No (%)		
Positive health self-assessment—being in better health than other people of the same age and gender (physical health)	491	34.00	66.00		
Not having any chronic diseases or ailments diagnosed by a doctor (physical health)	499	66.10	33.90		

**Table 2:** The relationship between work–life balance and physical health controlled by socio-demographic variables. Logistic regression analysis results.

	Positive Health Self-Assessment—Being in Better Health than Other People of the Same Age and Gender		Not Having Any Chronic Diseases or Ailments Diagnosed by a Doctor	
	Sig.	Exp(B)	Sig.	Exp(B)
2003 (n = 423)				
Work–life balance	-	-	-	-
Gender (1—female, 2—male)	-	-	-	-
Age	0.031	1.026	0.000	0.912
Education (1—lower than university, 2—university)	0.006	2.835		
Professional status: owner (ref. specialist)	-	-	-	-
Professional status: manager (ref. specialist)	-	-	-	-
Marital status—married (ref. single)	-	-	-	-
Marital status—divorced (ref. single)	-	-	-	-
Marital status—widowed (ref. single)	-	-	-	-
Having children up to 18 living in the household	-	-	-	-
Working time (in hours)	-	-	-	-
	Chi-square	Sig.	Chi-square	Sig.
Hosmer and Lemeshow Test	3.538	0.896	7.133	0.522
2013 (n = 473)				
Work–life balance	0.000	1.774	0.038	1.315
Gender (1—female, 2—male)	0.002	1.943	0.035	1.562
Age			0.000	0.958
Education (1—lower than university, 2—university)	-	-	-	-
Professional status: owner (ref. specialist)	0.042	1.748	-	-
Professional status: manager (ref. specialist)	-	-	-	-
Marital status—married (ref. single)	-	-	-	-
Marital status—divorced (ref. single)	-	-	-	-
Marital status—widowed (ref. single)	-	-	-	-
Having children up to 18 living in the household	0.031	0.548	-	-
Working time (in hours)	-	-	-	-
	Chi-square	Sig.	Chi-square	Sig.
Hosmer and Lemeshow Test	4.557	0.804	5.947	0.653

- There is no significant relationship.

**Table 3:** Relationship between work–life balance and the dimension of occupational stress: (mental health) controlled by socio-demographic variables. Results of multiple linear regression analysis.

	Satisfaction and Peace of Mind		Dynamism		Peace and Energy	
	Standardized Beta Coefficient	Significance	Standardized Beta Coefficient	Significance	Standardized Beta Coefficient	Significance
2003 (n = 441)						
Work–life balance	0.214	0.000	-	-	0.219	0.000
Gender (1—female, 2—male)	0.187	0.000	-	-	0.198	0.000
Age	0.092	0.049	-	-	-	-
Education (1—lower than university, 2—university)	-	-	-	-	-	-
Professional status: owner (ref. specialist)	-	-	-	-	-	-
Professional status: manager (ref. specialist)					0.141	0.000
Marital status—married (ref. single)	-	-	-	-	-	-
Marital status—divorced (ref. single)	-	-	−0.099	0.038	-	-
Marital status—widowed (ref. single)	-	-	-	-	-	-
Having children up to 18 living in the household	0.121	0.010	-	-	-	-
Working time (in hours)	-	-	-	-	-	-
Adjusted R Square	0.082		0.008 ^1^		0.093	
2013 (n = 469)						
Work–life balance	0.294	0.000	-	-	0.305	0.000
Gender (1—female, 2—male)	0.169	0.000	-	-	-	-
Age	0.147	0.001	−0.184	0.000	-	-
Education (1—lower than university, 2—university)	-	-	-	-	−0.100	0.025
Professional status: owner (ref. specialist)	-	-	-	-	-	-
Professional status: manager (ref. specialist)	-	-	-	-	-	-
Marital status—married (ref. single)	-	-	0.117	0.032	-	-
Marital status—divorced (ref. single)	-	-	0.191	0.000	-	-
Marital status—widowed (ref. single)	-	-	-	-	-	-
Having children up to 18 living in the household	-	-	-	-	-	-
Working time (in hours)	-	-	−0.104	0.024	-	-
Adjusted R Square	0.120		0.035		0.092	

^1^ model is not significant. - There is no significant relationship.

**Table 4:** Determinants of work–life balance. Results of multiple linear regression analysis.

	2003		2013	
	Beta	Sig.	Beta	Sig.
Gender (1—female, 2—male)	-	-	-	-
Age	-	-	-	-
Education (1—lower than university, 2—university)	0.160	0.003	-	-
Marital status—married (ref. single)	-	-	-	-
Marital status—divorced (ref. single)	-	-	-	-
Marital status—widowed (ref. single)	-	-	-	-
Size of household	-	-	-	-
Having children up to 18 living in the household	-	-	-	-
Professional status: owner (ref. specialist)	-	-	−0.130	0.005
Professional status: manager (ref. specialist)	-	-	-	-
Being on holiday no less than 7 days during the last 12 months	0.188	0.001	0.129	0.006
Doing extra paid work				
Willingness to change work	−0.180	0.001		
The sector where the given person is employed: private (ref. public)	-	-	-	-
The sector where the given person is employed: other (ref. public)		-	-	-
Working time (in hours)	−0.152	0.006	−0.228	0.000
Orientation towards success and career	-	-	0.101	0.041
Self-reliance	-	-	0.102	0.038
Protestant Work Ethic	0.128	0.025	-	-
Assessment of the importance of family and children	-	-	-	-
Assessment of the importance of occupation and work	−0.168	0.003	−0.163	0.001
Assessment of the importance of spare time and rest	0.255	0.000	0.141	0.007
Assessment of the importance of friends and acquaintances		-	-	-
Assessment of the importance of relatives		-	-	-
Assessment of the importance of religion and church		-	-	-
Assessment of the importance of politics and public life		-	-	-
Assessment of the importance of neighbours	0.145	0.013		
Assessment of the importance of health			0.150	0.004
Adjusted R Square	0.253		0.208	

- There is no significant relationship.

## Data Availability

The data presented in this study are available on request from the corresponding author. The data are not publicly available.

## References

[1] Davis S.N., Tuttle J.D. (2017). Context, Opportunity, and Demands: Satisfaction with Work-Life Balance in 26 Countries. J. Comp. Fam. Stud..

[2] (2016). European Quality of Life Survey. https://www.eurofound.europa.eu/data/european-quality-of-life-survey.

[3] Drygas W., Wojtyniak B., Drygas W., Słońska Z. (2017). Sytuacja zdrowotna ludności Polski na tle innych krajów [Health Status of Polish Population Comparing to Other Countries]. Jak Poprawić stan Zdrowia I Wpływać na Redukowanie Nierówności Społecznych w Zdrowiu w Społecznościach Lokalnych [How to Improve Health State and Have an Impact on the Reduction of Social Inequalities in Health in Local Communities].

[4] Wojtyniak B., Stokwiszewski J., Goryński P., Trochonowicz A., Zdrojewski T., Rabczenko D., Wojtyniak B., Goryński P. (2017). Długość życia i umieralność ludności Polski [Life Expectancy and Mortality of the Population of Poland]. Sytuacja Zdrowotna Ludności Polski i jej Uwarunkowania 2020 [Health Status of Polish Population and Its Determinants].

[5] Rantanen J., Kinnunen U., Mauno S., Tillemann K., Kaiser S., Ringlstetter M.J., Eikhof D.R., Pina e Cunha M. (2011). Chapter 2 Introducing Theoretical Approaches to Work-Life Balance and Testing a New Typology Among Professionals. Creating Balance?.

[6] Bell A.S., Rajendran D., Theiler S. (2012). Job Stress, Wellbeing, Work-Life Balance and Work-Life Conflict Among Australian Academics. E J. Appl. Psychol..

[7] Greenhaus J.H., Beutell N.J. (1985). Sources of conflict between work and family roles. Acad. Manag. Rev..

[8] Greenhaus J.H., Collins K.M., Shaw J.D. (2003). The relation between work–family balance and quality of life. J. Vocat. Behav..

[9] Netemeyer R.G., Boles J.S., McMurrian R. (1996). Development and validation of work-family conflict scales. J. Appl. Psychol..

[10] Frone M.R., Russel M., Cooper M.L. (1992). Antecedents and Outcomes of Work-Family Conflict: Testing a Model of the Work-Family Interface. J. Appl. Psychol..

[11] Grzywacz J.G., Marks N.F. (2000). Reconceptualizing the Work-Family Interface: An Ecological Perspective on the Correlates of Positive and Negative Spillover Between Work and Family. J. Occup. Health Psychol..

[12] Van Steenbergen E.F., Ellemers N. (2009). Is Managing the Work-Family Interface Worthwhile? Benefits for Employee Health and Performance. J. Organ. Behav..

[13] Lazarus R.S., Folkman S. (1984). Stress, Appraisal, and Coping.

[14] Titkow A. (1993). Stres i życie Społeczne. Polskie Doświadczenia [Stress and Social Life. Polish Experience].

[15] Demerouti E. (2018). Integrating Individual Strategies in the Job Demands-Resources Theory. IBR.

[16] Heikkilä K., Nyberg S.T., Fransson E.I., Alfredsson L., De Bacquer D., Bjorner J.B. (2012). Job Strain and Tobacco Smoking: An Individual-Participant Data Meta-Analysis of 166 130 Adults in 15 European Studies. PLoS ONE.

[17] Stubbs B., Veronese N., Vancampfort D., Prina A.M., Lin P., Tseng P.-T., Evangelou E., Solmi M., Kohler C., Koyanagi A. (2017). Perceived stress and smoking across 41 countries: A global perspective across Europe, Africa, Asia and the Americas. Sci. Rep..

[18] Hill D., Conner M., Clancy F., Moss R., Wilding S., Bristow M., O’Connor D.B. (2022). Stress and eating behaviours in healthy adults: A systematic review and meta-analysis. Health Psychol. Rev..

[19] Choi E., Kimb J. (2017). The association between work–life balance and health status among Korean workers. Work.

[20] Frone M.R., Russel M., Cooper M.L. (1997). Relation of work-family conflict to health outcomes: A four-year longitudinal study of employed parents. J. Occup. Organ. Psychol..

[21] Ioannidi D.-E., Nikolatou I., Sioula E., Galanakis M., Chrousos G.P., Darviri C. (2016). The Implications of the Conflict between Work and Family in Strain Levels: A Review Paper. Psychology.

[22] Janzen B.L., Muhajarine N., Kelly I.W. (2007). Work-Family Conflict, and Psychological Distress In Men And Women Among Canadian Police Officers. Psychol. Rep..

[23] Kafestios K. (2007). Work-family conflict and its relationship with job satisfaction and psychological distress: The role of affect at work and gender. HLP.

[24] Leineweber C., Baltzer M., Magnusson Hanson L.L., Westerlund H. (2012). Work–family conflict and health in Swedish working women and men: A 2-year prospective analysis (the SLOSH study). Eur. J. Public Health.

[25] Losoncz I., Bortolotto N. (2009). Work-life balance: The experiences of Australian working mothers. J. Fam. Stud..

[26] Härter G.R., Toivanen S., van Diepen C., Guimarães JM N., Camelo L.V., Juvanho L.L., Aquino E.M., Chor D. (2016). Work–Family Conflict and Self-Rated Health: The Role of Gender and Educational Level. Baseline Data from the Brazilian Longitudinal Study of Adult Health (ELSA-Brasil). IJBM.

[27] Winter T., Roos E., Rahkonen O., Martikainen P., Lahelma E. (2006). Work–Family Conflicts and Self-Rated Health Among Middle-Aged Municipal Employees in Finland. IJBM.

[28] Kim Y.-M., Cho S.-I. (2017). Work-Life Imbalance and Musculoskeletal Disorders among South Korean Workers. Int. J. Environ. Res. Public Health.

[29] Ejlertsson L., Heijbel B., Ejlertsson G., Andersson I. (2018). Recovery, work-life balance and work experiences important to self-rated health: A questionnaire study on salutogenic work factors among Swedish primary health care employees. Work.

[30] Baka Ł. (2013). Zależności między konfliktami praca–rodzina i rodzina–praca a zdrowiem pielęgniarek Buforujący efekt wsparcia społecznego. [Relationships between work–family and family–work conflicts and health of nurses buffering effects of social support]. Med. Pr..

[31] Chirkowska-Smolak T. (2008). Równowaga Między pracą a życiem osobistym. [Balance between work and private life]. Ruch. Praw. Ekon. Socjol..

[32] Rasmus P., Marcinkowska W., Cieleban N., Lipert A. (2020). Obciążenie pracą i radzenie sobie ze stresem a stan zdrowia pracowników systemu państwowego ratownictwa medycznego w kontekście work–life balance. [Workload and coping with stress and the health status of emergency medical Staff in the context of work–life balance]. Med. Pr..

[33] Warren T. (2015). Work–life balance/imbalance: The dominance of the middle class and the neglect of the working class. Br. J. Sociol..

[34] Bansal N., Agarwal U.A. (2017). Exploring Work-Life Balance among Indian Dual Working Parents. A Qualitative Study. J. Manag. Res..

[35] Buchheit S., Dalton D.W., Harp N.L., Hollingsworth C.W.A. (2016). Contemporary Analysis of Accounting Professionals’ Work-Life Balance. Acc. Horiz..

[36] Dhuru P. (2016). Study on Work Life Balance of Married Women in Banking Sector in Mumbai. IJRCM.

[37] Direnzo M.S., Greenhaus J.H., Weer C.H. (2015). Relationship between protean career orientation And work–life balance: A resource perspective. J. Organ. Behav..

[38] Haider S., Jabeen S., Ahmad J. (2018). Moderated Mediation between Work Life Balance and Employee Job Performance: The Role of Psychological Wellbeing and Satisfaction with Coworkers. JWOP.

[39] Kakkar J., Bhandari A. (2016). A Study on Work-Life Balance in the Indian Service Sector from a Gender Perspective. IUP J. Organ. Behav..

[40] Kaliannan M., Perumal K., Dorasamy M. (2016). Developing a work-life balance model towards improving job satisfaction among medical doctors across different generations. JDA.

[41] Malhotra J., Wong E., Thind A. (2018). Canadian family physician job satisfaction Is it changing in an evolving practice Environment? An analysis of the 2013 National Physician Survey database. BMC Fam. Pract..

[42] Drew N., Datta D., Howieson J. (2015). The Holy Grail: Work–Life Balance in the Legal. UNSW Law J..

[43] Mellner C., Aronsson G., Kecklund G. (2014). Boundary Management Preferences, Boundary Control, and Work-Life Balance among Full-Time Employed Professionals in Knowledge-Intensive, Flexible Work. Nord. J. Work Life Stud..

[44] Nilsson M., Blomqvist K., Andersson I. (2017). Salutogenic resources in relation to teachers work-life balance. Work.

[45] Pasewark W.R., Viator R.E. (2006). Sources of Work-Family Conflict in the Accounting Profession. Behav. Res. Acc..

[46] Seierstad C., Kirton G. (2015). Having It All? Women in High Commitment Careers and Work–Life Balance in Norway. Gend. Work Organ..

[47] Seong J.Y. (2016). Person–Organization Fit, Family-Supportive Organization Perceptions, and Self-Efficacy Affect Work–Life Balance. Soc. Behav. Personal. Int. J..

[48] Shah R. (2017). Development of a Bi-directional and Multi-dimensional Measure of Work-life Balance. SAJM.

[49] Sommerlad H. (2016). “A pit to put women in”: Professionalism, work intensification, sexualisation and work–life balance in the legal profession in England and Wales. Int. J. Leg. Prof..

[50] Bendix R. (1975). Max Weber. Portret Uczonego. [Max Weber. An Intellectual Portrait].

[51] Lockwood D. (1969). The Blackcoated Worker. A Study in Class Consciousness.

[52] Mills C.W. (1965). Białe kołnierzyki. Amerykańskie Klasy Średnie [White Collar: The American Middle Classes].

[53] Weber M. (2010). Etyka Protestancka a Duch Kapitalizmu. [Protestant Ethics and the Spirit of Capitalism].

[54] Ossowska M. (2005). Socjologia Moralności. Zarys Zagadnień. [Sociology of Morality: Outline of Issues].

[55] Zdrenka M.T. (2003). Problem Uniwersalizacji Etosu mieszczańSkiego. [The Problem of Universalization of the Protestant Ethos].

[56] Arslan M. (2000). A cross-cultural comparison of British and Turkish managers in terms of Protestant work ethic characteristics. BUETFV.

[57] Arslan M., Chapman M. (2001). Work ethic values of practising catholic Irish and protestant British managers. Irish J. Manag..

[58] Zulfikar Y.F. (2012). Do Muslims Believe More in Protestant Work Ethic than Christians? Comparison of People with Different Religious Background Living in the US. J. Bus..

[59] Bauman Z., Jawłowska A., Kempny M. (2005). Konsumując życie [Consuming Life]. Konsumpcja istotny Wymiar Globalizacji Kulturowej [Consumption Important Dimension of Cultural Globalisation].

[60] Bell D. (1994). Kulturowe Sprzeczności Kapitalizmu [The Cultural Contradictions of Capitalism].

[61] Bylok F., Jawłowska A., Kempny M. (2005). Model społeczeństwa konsumpcyjnego i jego zastosowanie na początku XXI wieku. [The Model of Consumer Society and its Application at the Beginning of 21st Century.]. Konsumpcja Istotny Wymiar Globalizacji Kulturowej [Consumption Important Dimension of Cultural Globalisation].

[62] Campbell C. (1983). Romanticism and The Consumer Ethic: Intimations of a Weber-style Thesis. Sociol. Anal..

[63] Boniface D.R., Cottee M.J., Neal D., Skinner A. (2001). Social and demographic factors predictive of change over seven years in CHD-related behaviours in men aged 18 ± 49 years. Public Health.

[64] Bourdieu P. (2005). Dystynkcja. Społeczna Krytyka Władzy Sądzenia [Distinction: A Social Critique of the Judgement of Taste].

[65] Crawford R. (1980). Healthism and the medicalization of everyday life. Int. J. Health Serv..

[66] Jones I.R., Papacosta O., Whincup P.H., Wannamethee S.G., Morris R.W. (2011). Class and lifestyle ‘lock-in’ among middle-aged and older men: A Multiple Correspondence Analysis of the British Regional Heart Study. Sociol. Health Illn..

[67] Tomlinson M. (2003). Lifestyle and Social Class. Eur. Sociol. Rev..

[68] Warde A. (2006). Cultural Capital and the Place of Sport. Cult. Trends.

[69] Domański H. (1994). Społeczeństwa Klasy Średniej [The Middle Class Societies].

[70] Domański H. (1999). Klasa średnia. [Middle Class]. Encyklopedia Socjologii [Encyclopedia of Sociology].

[71] Domański H. (2002). Polska Klasa Średnia [The Polish Middle Class].

[72] Drozdowski R. (1998). Kontrowersje wokół klasy średniej w Polsce lat dziewięćdziesiątych. [The controversies about the middle class in Poland of the nineties]. Kult. Spolecz.

[73] Gdula M., Sadura P., Gdula M., Sadura P. (2013). Style życia jako rywalizujące uniwersalności. [Lifestyles as Competing Universalities]. Style Życia i Porządek Klasowy w Polsce [Lifestyles and Class Order in Poland].

[74] Kurczewski J. (1995). Stare i nowe klasy średnie w Polsce [Old and New Middle Classes in Poland]. Ludzie i instytucje. Stawanie się ładu Społecznego. Pamiętnik IX Ogólnopolskiego Zjazdu Polskiego Towarzystwa Socjologicznego [Peoples and Institutions. Formation of a Social Order. The Diary of IX Polish Sociological Congress].

[75] Leszkowicz-Baczyński J. (2007). Klasa Średnia w Polsce? Sytuacja Pracy, Mentalność Wartości [Middle Class in Poland? Work Situation, Mentality, Values].

[76] Mokrzycki E. (1994). Nowa klasa średnia? [New Middle Class?]. Stud. Socjol..

[77] Sadura P., Gdula M., Sadura P. (2013). Wielość w jedności: Klasa średnia i jej zróżnicowanie. [Plurality in Unity: Middle Class and its Differentiation]. Style Życia i Porządek Klasowy w Polsce [Lifestyles and Class Order in Poland].

[78] CBOS (2011). Mania Kupowania, Czyli o Orientacjach Konsumenckich Polaków. Komunikat z Badań. [Shopping Mania, i.e., about the Consumer Orientations of Poles. Research Report].

[79] CBOS (2014). Zachowania Żywieniowe Polaków. Komunikat z Badań [Nutritional Behaviour of Poles. Research Report].

[80] CBOS (2018). Aktywność Fizyczna Polaków. Komunikat z Badań [Physical Activity of Poles. Research Report].

[81] CBOS (2019). Jak Zdrowo Odżywiają się Polacy. Komunikat z Badań. [How Healthy Do Poles Eat. Research Report].

[82] CBOS (2019). Postawy Wobec Palenia Papierosów. Komunikat z Badań. [Attitudes towards Cigarettes Smoking. Research Report].

[83] Derczyński W., Falkowska M. (1997). Postawy i zachowania konsumenckie [Consumer Attitudes and Behaviour]. Praca, Wartości Zakupy... O stylach Życia Polaków. [Work, Values, Shopping… about Lifestyles of Poles].

[84] Domański H. (2001). Klasy średnie w Polsce a wybrane aspekty etosu protestanckiego. [Polish Middle Classes and Some Aspects of the Protestant Ethics in Poland]. Kult. Spolecz.

[85] Borkowska S. (2010). Równowaga między pracą a życiem pozazawodowym. [The Work-Life Balance]. Acta Univ. Lodz. Folia Oecon..

[86] Urząd Statystyczny w Warszawie (2001). Panorama Dzielnic Warszawy w 2000 Roku [Panorama of Warsaw Districts in 2000].

[87] Główny Urząd Statystyczny (2002). Rocznik Statystyczny Województwa Mazowieckiego 2002 [Statistical Yearbook of Mazowieckie Voivodships 2002].

[88] Urząd Statystyczny w Warszawie (2012). Panorama Dzielnic Warszawy 2011 [Panorama of Warsaw Districts in 2011].

[89] Barometr Warszawski 2013 [Warsaw Barometer 2013]. https://um.warszawa.pl/waw/warszawa-w-liczbach/barometr-warszawski6.

[90] Alexopoulos E.C., Geitona M. (2009). Self-Rated Health: Inequalities and Potential Determinants. Int. J. Environ. Res. Public Health.

[91] Dziankowska-Zaborszczyk E., Ciabiada B., Maniecka-Bryła I. (2014). Samoocena stanu zdrowia jako predyktor umieralności przedwczesnej [Self-Rated Health as a Premature Mortality Predictor]. Probl. Hig. I Epidemiol..

[92] Mossey J.M., Shapiro E. (1982). Self-rated health: A predictor of mortality among the elderly. Am. J. Public Health.

[93] Wróblewska W. (2010). Stan zdrowia w Polsce rola czynników ekonomiczno-społecznych i stylu życia. Ocena na podstawie wskaźnika SRH i PAR. [State of Health in Poland The Role of Economic and Social Factors and Lifestyle. Estimation Based on SRH and PAR Indicators]. Zesz. Nauk. Inst. Stat. I Demogr. SGH.

[94] Cullati S. (2014). The influence of work-family conflict trajectories on self-rated health trajectories in Switzerland: A life course approach. Soc. Sci. Med..

[95] Widerszal-Bazyl M. (2001). Podręcznik do Testu “Stres w Pracy”. Polska Adaptacja skrÓconej Wersji Occupational Stress Indicator OSI 2. [Textbook to the Test “Stress in Work”. Polish Adaptation of Shortened Version of Occupational Stress Indicator OSI 2].

[96] Domański H., Dukaczewska A. (1994). Orientacje indywidualistyczne w Polsce. [Individualistic Orientations in Poland]. Kult Spolecz.

[97] Domański H., Dukaczewska A., Domański H., Rychard A. (1997). Samodzielność i chęć polegania na sobie. [Independence and the desire to rely on oneself]. Elementy Nowego Ładu [The Elements of a New Order].

[98] Firkowska–Mankiewicz A. (1999). Zdolnym być...: Kariery i Sukces Życiowy Warszawskich Trzydziestolatków. [Being Talented: Careers and Life Success of Thirty-Years-Old Varsovians].

[99] Swadźba U., Bylok F., Swadźba U. (2014). Od świata pracy do świata konsumpcji. Analiza socjologiczna systemu wartości. *[From the World of Work to the World of Consumption. Sociological Analysis of Values]*. Między pracą a konsumpcją. “Co decyduje o miejscu człowieka w dzisiejszym społeczeństwie?” [Between Work and Consumption. What Does Decide about the Man Place in the Modern Society?].

[100] Leszkowicz-Baczyński J., Zawadzka A.M., Górnik-Durose M. (2010). Konsumpcja klasy średniej w Polsce. Czy konsumowanie wyznacza etos. [Does The Consumption Define Ethos?]. Życie w Konsumpcji, Konsumpcja w Życiu. Psychologiczne Ścieżki wspóŁzależności. [Life in Consumption, Consumption in Life. Psychological Paths of Interdependence].

[101] Bharath M.J., Crutsinger C., Reynolds J.S., Dotter T.-V., Thozhur S., Baum T., Devine F.G. (2009). An empirical study of the work attitudes of generation Y college student in the USA: The case of hospitality and merchandising undergraduate majors. J. Serv. Res..

[102] Reisewitz T.H., Iyer R. (2009). Differences in generation X and Generation Y: Implications for the organization and marketers. Mark. Manag. J..

[103] Meriac J.P., Woehr D.J., Banister C. (2010). Generational Differences in Work Ethic: An Examination of Measurement Equivalence Across Three Cohorts. J. Bus. Psychol..

[104] Dymek J. (2015). Żegnaj klaso średnia, witaj prekaria cie [Goodbye the Middle Class, Welcome the Precariat]. Z Guyem Standingiem rozmawia Jakub Dymek. Kryt. Polit..

[105] Standing G. (2014). Prekariat [The Precariat].

[106] Sutowski M. (2015). Klasa średnia?: A może po prostu “młodzi, wykształceni, z wielkich miast”? [Middle Class?: And Maybe Simply: Young, Educated from the Big Cities?]. z Kazimierzem Frieske rozmawia Michał Sutowski. Kryt. Polit..

[107] Temin P. (2017). The Vanishing Middle Class. Prejudice and Power in a Dual Economy.

[108] Koralewicz J., Ziółkowski M. (2003). Mentalność Polaków. [Mentality of Poles].

[109] Frąckowiak-Sochańska M. (2022). Emocjonalna praca graniczna jako kategoria użyteczna w badaniach relacji pomiędzy pracą zawodową a życiem prywatnym w czasie pandemii COVID -19. [Emotional Boundary Labor as a Useful Category for the Studies of the Relations Between Professional Work and Private Life During COVID-19 Pandemic]. Stud. Socjol..

[110] Neidlinger S.M., Felfe J., Schübbe K. (2023). Should I Stay or Should I Go (to the Office)?—Effects of Working from Home, Autonomy, and Core Self–Evaluations on Leader Health and Work–Life Balance. Int. J. Environ. Res. Public Health.

[111] Jones J.S., Murray S.R., Tapp S.R. (2018). Generation Differences in the Workplace. JBD.

[112] Schilpzand A., de Jong E. (2021). Work ethic and economic development: An investigation into Weber’s thesis. Eur. J. Polit. Econ..

